# Toxicity of secondary metabolites of *Paenarthrobacter nicotinovorans*

**DOI:** 10.17912/micropub.biology.000923

**Published:** 2023-09-19

**Authors:** Justyna Kakol, Mainor Vang, Drew Sausen, Tracey Steeno, Angelo Kolokithas

**Affiliations:** 1 Northeast Wisconsin Technical College, Green Bay, Wisconsin, United States

## Abstract

In a previous study, *Paenarthrobacter nicotinovorans* was isolated and screened for antimicrobial activity. Many promising antibiotic candidates are not ultimately used because of toxicity. After screening secondary metabolites for antimicrobial activity, they were screened for cytotoxicity *in vitro* in the human HeLa cell line. The results show that there is no detectable cytotoxicity in HeLa cells.

**Figure 1. Toxicity of Paenarthrobacter nicotinovorans secondary metabolites on HeLa cells:**
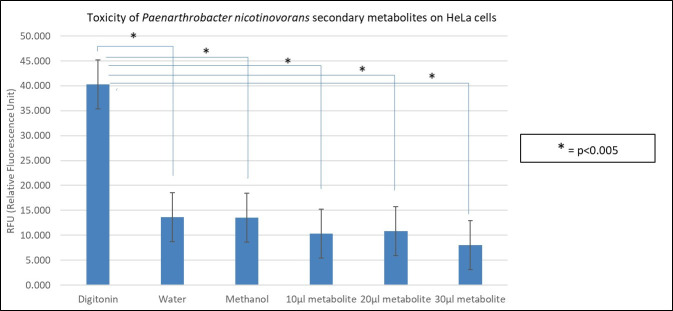
Relative fluorescence units (RFU) measured for the following samples: positive control (digitonin), solvent control (methanol), negative control (water), and three different concentrations of the secondary metabolite (10 µL, 20 µL, and 30 µL). The error bars represent standard error from the mean and the program used to calculate p values was Microsoft Excel using a t-test. The p values concerning the difference between digitonin and water was 6.92 x 10 ^-6^ , digitonin and methanol was 3.61 x 10 ^-6^ , digitonin and 10μl of metabolite was 3.34 x 10 ^-6^ , digitonin and 20μl of metabolite was 7.60 x 10 ^-6^ , and digitonin and 30μl of metabolite was 6.48 x 10 ^-6^ .

## Description

Upon finding that the secondary metabolites of *Paenarthrobacter nicotinovorans* were displaying antimicrobial activity (Kakol et al., 2023) , the toxicity of the metabolites were tested *in vitro* . The CytoTox-Fluor™ Cytotoxicity Assay was utilized to test the cytotoxicity of increasing amounts of the secondary metabolites as compared to the toxicity of digitonin, methanol, and water ( Figure 1 ). Increasing the amount of secondary metabolites did not result in increased toxicity as compared to the digitonin control and were more like the negative controls.

Though *Paenarthrobacter nicotinovorans* is described in the literature being involved in nicotine metabolism, its antimicrobial activity has only been tested by the production of silver nanoparticles. (Huq and Akter, 2021) One limitation of this experiment is only one measure of cytotoxicity was utilized, though there are many more available on the market to identify different types of toxicity. Further studies should utilize these measures to identify toxicity that this assay may have missed. Nonetheless, *Paenarthrobacter nicotinovorans* represents a good candidate for further antimicrobial development as it seems not to display toxicity in Hela cells.

## Methods

*Cell culture*

Hela cells (ATCC #CCL-2) were maintained in Dulbecco's Modified Eagle Medium (DMEM) supplemented with 10% fetal bovine serum (FBS) and 1x Penicillin-Streptomycin-Neomycin (PSN).

*Toxicity testing*

The toxicity of the secondary metabolites from the *Paenarthrobacter nicotinovorans* bacteria to the HeLa cells was measured by using a commercial CytoTox-Fluor™ Cytotoxicity Assay (Promega Catalog #G9260) according to the manufacturer’s instructions. The positive control was 300ug/ml Digitonin (Orczyk et al, 2017) (Invitrogen, BN2006). Methanol (Sigma, 67-56-1) and DI H2O were used as vehicle and negative controls, respectively. The extract of *Paenarthrobacter nicotinovorans* (10mg/ml) was used at various concentrations in the toxicity experiments, and various incubation times were used to determine if concentration or incubation time increased toxicity. Ten thousand Hela Cells per well were used in a 96-well plate and the fluorescence measurements were obtained using the Varioskan™ LUX (ThermoFischer) multimode microplate reader with the rhodamine-110 (485nmEx/520Em) filter set.

*Statistics*

Microsoft Excel was used to analyze the data. Student T-tests were utilized to determine the level of significance (p values). All experiments and samples were run in triplicate.

## References

[R1] Huq MA, Akter S (2021). Bacterial Mediated Rapid and Facile Synthesis of Silver Nanoparticles and Their Antimicrobial Efficacy against Pathogenic Microorganisms.. Materials (Basel).

[R2] Kakol J, Vang M, Sausen D, Steeno T, Kolokithas A (2023). Antimicrobial activity of Paenarthrobacter nicotinovorans.. MicroPubl Biol.

[R3] Orczyk M, Wojciechowski K, Brezesinski G (2017). Disordering Effects of Digitonin on Phospholipid Monolayers.. Langmuir.

